# Behavior in Free-Living American Black Bear Dens: Parturition, Maternal Care, and Cub Behavior

**DOI:** 10.3390/ani10071123

**Published:** 2020-07-01

**Authors:** Lynn L. Rogers, Linda McColley, Janet Dalton, Jim Stroner, Douglas Hajicek, Adam Partin, Gordon M. Burghardt

**Affiliations:** 1 Wildlife Research Institute, Ely, MN 55731, USA; lrogers@bearstudy.org (L.L.R.); linda@bearstudy.org (L.M.); janetdalton02@gmail.com (J.D.); jim@stronerwildlife.com (J.S.); 2 White Wolf Entertainment, Inc., Minneapolis, MN 55448, USA; dhajicek@usinternet.com; 3 Department of Psychology, University of Tennessee, Knoxville, TN 37996, USA; apartin3@vols.utk.edu; 4 Departments of Ecology and Evolutionary Biology, University of Tennessee, Knoxville, TN 37996, USA

**Keywords:** American black bear, *Ursus americanus*, hibernation, denning behavior, maternal behavior, behavioral ontogeny, play

## Abstract

**Simple Summary:**

We report here some of the major findings on the behavior of black bear mothers and cubs in their dens in the wild, based on observations in the state of Minnesota, USA. Wild female bears were outfitted with radio collars and their dens located as they prepared for hibernation in the fall. Cameras were installed in the dens and events in the den recorded until they and their cubs finally abandoned their dens in the spring. Although most reports of black bear cub behavior have been post den emergence, we provide intimate details of their birth, maternal behavior, and the development of the cubs from birth to emergence. Yearling cubs from the previous year sometimes remained with their mother for a second year. We discovered many aspects of mother, cub, and yearling behavior previously unknown and some of which contradict claims in the literature. Den cams are an important means of observing secretive behavior in settings previously impossible to observe unobtrusively.

**Abstract:**

Denning behavior has long remained the least observed aspect of bear behavior. During 2010–2013, we used webcams, microphones, the internet, and 14,602 h of archived video to document the denning behaviors of two adult wild black bears (*Ursus americanus*) as they gave birth and cared for four litters through six winters in northeastern Minnesota. Observations included types of dens, labor, pre-parturient genital swelling, birthing positions, post-partum vocalizations, mothers removing amniotic tissues and warming newborn cubs in sub-freezing temperatures, frequency of nursing, cubs establishing nipple order, yearlings suckling, the ingestion of snow and icicles, the ingestion of foot pads, urination and defecation in latrine areas, toilet-licking, eye opening, reciprocal tongue-licking, play, rapid eye movement (REM) sleep and possible dreaming, and reactions to wildlife intruders. The use of this new method for observing natural bear dens allowed the identification of many behaviors undescribed for any species of wild bear in dens. We also discuss the need for future studies and how the depth and duration of black bear hibernation varies with body condition and geographic region.

## 1. Introduction

The behavior of bears in natural dens has long remained the least observed aspect of bear behavior. Prior to the invention of webcams in the 1990s, studies of denning behavior generally were limited to pre-denning and post-denning behaviors and the types of dens selected [1,2]. Attempts to observe black bear (*Ursus americanus*) behaviors by visiting winter dens were hampered by observer effects and den abandonments [3,4,5,6,7,8,9,10,11]. The absence of observations of birthing behavior led to a long-held belief that hibernating black bears “doze” through parturition [12]. With the advent of webcam technology, solar panels, and methods for tracking bears to natural den sites, we now have the means to effectively spy on the most private aspects of life in these animals.

Black bears in northeastern Minnesota typically produce their first litters at 3–8 years of age in January—the coldest month of the year [11,13]. Mothers care for their one to four cubs for 16–17 months until they mate again in May or June, giving birth every other January. Black bear cubs are nearly hairless and smaller, relative to the mother’s size, than other placental mammals [14]. They average only 364 g at birth [15]) while mothers can weigh over 100 kg. These extremely altricial cubs require constant care in their hidden world of dens. Previous observations of cub behavior and its development have begun after cubs emerged from the den, typically at 2.5 to 3 months of age [16]. Descriptive accounts of bear behavior are essential to ethological analysis and comparative behavior [17] and this work provides methods and incentives to carry out such studies on other species and populations.

Here, we summarize birthing behaviors, early cub care, and other undisturbed behaviors of black bears that we observed using the internet and 24/7 webcam video recording in northeastern Minnesota during 2010–2013. Den data include: dimensions and types of dens and dates of ingress and egress. Pre-parturition data include genital swelling and den preparation. Parturition and post-parturition data include body position during birth, initial vocalizations, cleaning and warming cubs, the frequency of nursing, the establishment of nipple order, water intake, urination, defecation, fecal plugs, toilet-licking, suckling behavior, water intake, urination, eye opening, reciprocal tongue-licking, play, REM sleep and dreaming, reactions to small mammals and spiders in dens, and reactions to large mammals near dens.

Many behaviors described here have not previously been mentioned as occurring in dens, and have even been claimed not to occur [18]. Given that black bears are a common species, a game animal, a tourist draw, highly intelligent, potentially dangerous, and threatened in many parts of their historical and current range, more information on all aspects of their life history in the wild is needed. Furthermore, behavioral development in these highly altricial animals can be theoretically and comparatively important. Dogs, wolves, and related canids are the focus of much developmental research [19,20,21,22,23,24], while bears, although less social as adults than many other carnivores, have a far longer period of maternal care, and can provide tests of hypotheses deriving from research on canids.

## 2. Materials and Methods 

### 2.1. Study Area

The six dens observed in this study were located 17–24 km west-southwest of Ely, MN, USA and all were <3 km from 47°48′42.75′′ N, 92°06′36.5′′ W in the cool temperate northern Great Lakes region where boreal conifer forest and temperate deciduous forest overlap [25]. The growing season averages 118 days between mid-May and mid-September, and snow cover is typically present from November to April [11]. Few trees in the region have hollows large enough for dens [26], so dens are typically on or below ground with open entrances [11]. The study area has a scattering of old white pines (*Pinus strobus*) that are highly preferred as refuge trees by mothers with cubs in this region [26]. Among their other characteristics, these trees have strong, furrowed bark that cubs can safely climb [26]. We located the dens by locating radio-collared females. We measured dens (burrows and entrances) in terms of Length (L), Height (H), and Width (W) and collected feces after bears left their dens in spring. All dens were underground and were either constructed by the females, previously used dens, or natural formations. 

### 2.2. Subjects 

The subjects were two female black bears (*Ursus americanus*) and their cubs and included six denning events by the two females, Lily and Jewel, born in mid-January in 2007 and 2009, respectively. Data on the dens and dates of ingress and egress are in Table 1 and information on the females and their cubs is in Table 2. A total of 3 male (M) and 4 female (F) cubs were observed; some over two denning events.

### 2.3. Recording Methods

As part of a long-term study of black bear behavior, ecology, and physiology [11,27,28,29,30,31,32,33], we adapted Goodall’s [34] trust-based methods to black bears [32]. Using those methods, we radio-collared bears without using tranquilizers, accompanied them for 24–48 h at a time, and during 2010–2013, encased webcams and microphones in PVC pipes and in early winter (before 8 January), placed them in six dens occupied by mature females Lily and Jewel. We connected the webcams and microphones to solar-powered broadcast equipment 51–138 m from the dens and used cell phone technology to transmit the video (with sound) to South Africa, where WildEarth.tv archived it and streamed it live online from early winter until the bears emerged in spring. The specific equipment varied over the seasons as improvements became available, but all involved the analog to digital conversion of video and microphone input. For the 2013 dens, we used XTC Corp digital IP cameras. The camera system was designed by William Powers (Export, PA, USA), Jim Stroner (Hopkins Crossroad, MN, USA), and Douglas Hajicek (Anoka, MN, USA), and it consisted of a Video Encoder, Digi TransPort WR21 Cellular Router, Raptor Control Board, a Video Switcher Board, and a Cellular Verizon 4G Upload Connection.

### 2.4. Behavior Analysis

The primary observational data were gleaned from the video records by LLR and LM with occasional alerts from the online webcam followers and checking by AP and GMB. The primary goal of this paper is to document the occurrence of specific behaviors, the dates on which they occurred, and the post-partum age of the cubs. We determined the sexes of newborn cubs from the male’s foreskin or the female’s vulva and identified individual cubs by overall fur color and the color patterns of the chest, muzzle, ears, forehead, and eyebrow area. The ability to identify specific individuals and behaviors was often obscured by bedding, body position, webcam alignment, and dirty camera lenses. Nevertheless, webcams provided a degree of visibility without a person present for months at a time. On our occasional visits to clean lenses or align webcams, the bears recognized our verbal routines and showed little sign of disturbance.

A total of 14,602 h of online video/audio were recorded and archived during 2010–2013. Additional quantitative screening was carried out by over 100 online viewers trained and supervised by JD to work singly or in pairs to cover each hour of the day and night to record when bears were active/inactive or when unusual behavior occurred. To determine the establishment of nipple order, we observed the individual use of nipples, as well as competition. To determine the frequency and duration of nursing, we listened for the distinctive hum of suckling. To determine temperatures (ambient and in open dens), we used data from the Ely, MN weather station 23 km 63° ENE of the study area, knowing that temperatures in open dens are 1–2 °C warmer than ambient [11]. For this paper, we supplemented webcam observations with incidental unpublished observations from LLR’s 50 years of black bear study, and these are noted. The massive quantitative activity records merit separate analysis; here, we focus on previously undocumented observations and developmental milestones.

## 3. Results

On our occasional visits to maintain the webcams, the bears recognized and accepted our presence. If the mothers were nursing cubs, as evidenced by the pulsing hum vocalization of suckling cubs [35], the mothers did not move. If the mothers had their heads tucked under their chests tending to cubs, they seldom looked up. No bear abandoned its den. If the mothers were active, we occasionally took heart rate estimates by observing the bears and, if lying still, counting hair twitches that correlated with heart rate. Counts were made for 20–30 s and converted to beats per minute. Given the camera placement and mother and cub position in the den, many of the following observations were based on only some of the dens and, in others, the date of the confirmation of developmental age are conservative.

### 3.1. Lily’s Four Dens

#### 3.1.1. Den Event 1

On 3 October 2009, 33-month-old Lily entered a shallow burrow that had previously been used by another bear. At 11:36 on 22 January 2010, three-year-old Lily gave birth to a single female (Hope). At 09:10 on 1 April, after being at the den for 180 days (5.9 mos.), Lily led 69-day-old Hope ~250 m to an old white pine.

#### 3.1.2. Den Event 2

**Figure 1:**
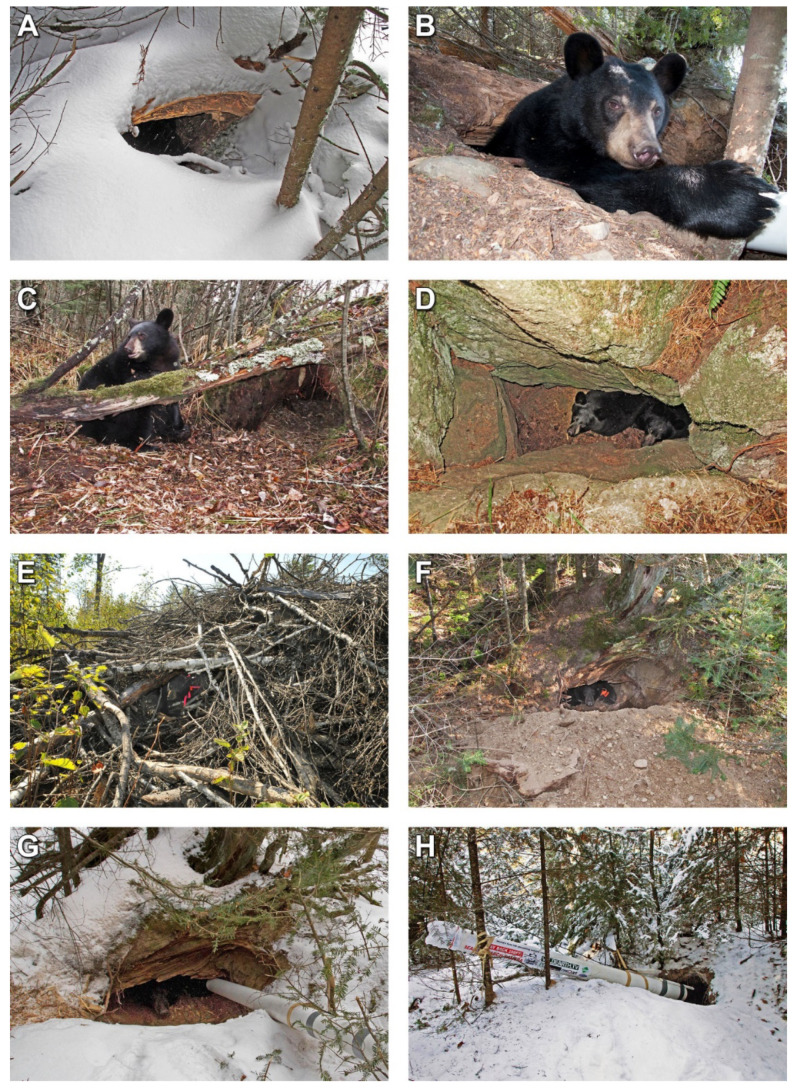
(**A**). Lily’s burrow den on 4 December 2009. (**B**). Lily at den 24 March 2010. The white tube extending into the den contains the webcam installed on 8 January 2010. (**C**). Nine-month-old Hope at the root mound den on 25 October 2010, showing vegetation that she and three-year-old Lily had raked into it for bedding. (**D**). Four-year-old Lily and nine-month-old Faith in the deep rock den 12 November 2011. (**E**). Five-year-old Lily in the slash pile den on 10 September 2012. (**F**). Two-year-old Jewel showing the dirt mound she excavated to enlarge her burrow den of the previous year on 22 October 2011. (**G**). Two-year-old Jewel in her enlarged burrow den with the webcam installed on 7 January 2012. (**H**). The burrow den of three-year-old Jewel with its throw mound and webcam on 23 December 2012. It was used by Jewel and her yearlings Fern and Herbie in the winter of 2012–2013.

On 19 October 2010, 45-month-old Lily and nine-month-old Hope arrived at a root mound where, years earlier, a cedar tree (*Thuja occidentalis*) had blown over, lifting roots and soil to create a large cavity. They immediately began raking ground vegetation into it for bedding (Figure 1C). At 13:51 and 15:02 on 21 January 2011, four-year-old Lily gave birth to Faith and Jason. On 8 April 2011, after being at the den for 171 days (~5.6 mos.), Lily led the 77-day-old cubs and her 14½-month-old yearling away from the den. Overnight, they moved 0.7 km to an old white pine. There, Jason received a bite to the head and died of a brain infection on 12 April, according to the University of Minnesota’s Veterinary Diagnostic Laboratory. Nearby patches of snow showed fresh coyote (*Canis latrans*) tracks. On 16 September 2011, 20-month-old Hope was killed by a hunter, leaving only Faith to den with Lily that fall.

#### 3.1.3. Den Event 3

On 20 October 2011, 57-month-old Lily and nine-month-old Faith began raking vegetation for bedding into a rock crevice 2.5 m deep (Figure 1D). On 24 March 2012, after being at the den only 156 days (5.1 months), Lily led 14-month-old Faith away from the den after an early 14-day warm spell (average 8.9 °C, range −7 to 25 °C) melted the snow, making it the shortest denning period in the study. 

#### 3.1.4. Den Event 4

On 31 August 2012, pregnant and lethargic with a heart rate of only 57 bpm, Lily bedded near a two-year-old slash pile where she remained and was found sleeping 2.2 m inside it on 10 September (Figure 1E). At 01:00 and 01:22 on 12 January, six-year-old Lily gave birth to Eli and Ellie. On 23 April, after being at the den for 235 days (7.7 mos.), Lily led her 101-day-old cubs away from the den after a late blizzard (56 cm of snow) and a week of temperatures as low as −23 °C. It was the longest denning period in the study. Snow depth at the time of their leaving was 66 cm, but temperatures as high as 4.4 °C and as low as −10 °C in the two previous days created a crust the family could walk on.

### 3.2. Jewel’s Two Dens

#### 3.2.1. Den Event 1

In mid-October 2011, 33-month-old Jewel enlarged the sandy burrow she had used as a den the previous winter, creating a large pile of soil (Figure 1F). The soil sloped down to the burrow that extended under the rotting wood of a large tree that had fallen decades earlier. She did not rake bedding into the den. The exact date of den ingress is uncertain. On 7 January 2012, we placed a webcam and microphone in the den (Figure 1G). At 07:22 and 08:40 on 22 January, three-year-old Jewel gave birth to Fern and Herbie. On 12 April, after being at the den for 173–183 days (5.7–6.0 mos.), Jewel led her 81-day-old cubs 160 m to a large old white pine.

#### 3.2.2. Den Event 2

On 8 October 2012, 45-month-old Jewel and 8½-month-old Fern and Herbie stopped foraging and began moving 1.2 km to a den site 0.86 km from their previous den in the same vein of easily dug sand. Overnight, they dug one of the biggest dens we have seen—a burrow 266 cm long with a terminal bowl-shaped chamber 72 cm in diameter and 58 cm from floor to ceiling. They threw fresh sand as far as 3.7 m out the entrance. She did not rake bedding into the den. On 23 December, we installed a webcam and microphone (Figure 1H). At 20:17 on 27 April 2013, after being at the den for 201 days (6.6 mos.), Jewel and her two yearlings moved 0.4 km to the base of a mature white pine. 

### 3.3. Pre-Parturition: Genital Swelling

In the weeks preceding parturition, the bears usually lay in ventrally recumbent positions that hid the genitalia. However, as early as 11 days before giving birth, both mothers were observed in positions that revealed greatly swollen vaginal openings and surrounding tissues.

### 3.4. Pre-Parturition: Labor

In 2010, Lily lay in positions that let us see her begin flexing her head muscles as she began clenching her teeth 21 h and 39 min before she gave birth to a single cub on 22 January 2010. She did the same 20 h and 43 min before she gave birth to two cubs on 21 January 2011. Twelve hours and 42 min before the birth in 2010, she slammed her body against the den wall six times in 4 min. In the 3 h before Jewel gave birth in 2012, her midsection was moving, possibly due to contractions. During that time, she occasionally licked her swollen genitals, emitting soft moans. During Lily’s final 31 min before parturition in 2010, she intermittently licked her swollen genitals without vocalizing (Video S1). Two minutes before parturition, Jewel doubled her breathing rate to nine breaths per minute.

### 3.5. Parturition

The two mothers squatted for at least six of their births (four litters). A seventh birth on 12 January 2013 was less visible. For that birth, Lily approached parturition lying on her back, bracing her feet against the ceiling of her slash pile den, but sat erect around the time of birth. Lily did not present such views prior to giving birth to her other two litters.

### 3.6. Initial Care of Cubs: Cleaning and Warming

Ground dens with open entrances, such as the six dens reported here, are only 1–2 °C warmer than the ambient air [11]. The four litters in this study were born into temperatures of +2, −8, −8, and −18 °C, respectively, and endured temperatures as low as −39 °C in the 24 h that followed.

Activities were sufficiently visible for three litters born in 2010 (Lily), 2011 (Lily), and 2012 (Jewel) to determine that the mothers began licking embryonic membranes from the firstborn cubs within 9, 16, and 85 s of parturition. On 22 January 2012 (−8 °C), Jewel licked her firstborn 77 times per minute for 6.5 min before assuming the ventrally recumbent warming position and placing the cub under her sparsely furred chest and belly. In that position, with her head tucked under her chest and her crown against the den floor, she continued licking the cubs dry while warming them with her breath. Her legs were against her sides, neck, and head, forming a chamber that minimized the loss of warm, humid air, as was also reported in Pennsylvania [9]. This warming position also minimized Jewel’s own surface area, heat loss, and water loss [36].

### 3.7. Nursing and Establishment of Nipple Order 

In the hours before milk let-down, Jewel was quiet when the cubs were quiet, but answered with grunts when the cubs squeaked and chirped using voices that were not fully developed. The pulsing hum of suckling [35], also squeaky at first, began 3 h and 1 min after Jewel gave birth to her first cub of her first litter (2012). For Lily’s litters, this suckling sound began 231 min after the birth of her first cub in 2010 but only 89 and 81 min, respectively, after the births of the first cub of her second and third litters in 2011 and 2013. At four days of age, Lily’s single cub engaged in 64 nursing sessions in 24 h with sessions averaging 2 min (range <1 to 22 min). Forty-four of the sessions were ≤1 min. 

The litters of two cubs born to Jewel in 2012 and to Lily in 2013 established peaceful and consistent nipple orders by 17 February and 15 April, respectively, which they continued after leaving their dens.

### 3.8. Initial Care of Cubs: Toilet-Licking

Most newborn mammals require external stimulation of the perineal region (toilet-licking) to induce micturition and defecation [37]. In the process, mothers ingest the urine and feces and avoid fouling their living quarters [37]. Lily and Jewel routinely did this, often in response to the cubs’ cries. Licking lasted 53 to 88 s in six timed observations between 24 January and 5 March (Video S2). We found no reference in the literature regarding toilet-licking by bears.

### 3.9. Suckling by Yearlings 

Yearlings Faith, Fern, and Herbie frequently suckled in dens despite the fact that their mothers’ mammary glands were flaccid and we saw no milk. On 19 February 2013 at 13:07:14, one of Jewel’s yearlings (Fern or Herbie) bawled long and loud to suckle and was finally allowed to do so at 13:18:31. In previous observations, in 1990, yearlings Mary and Gerry suckled in the den and after emergence, but we were unable to express milk from the flaccid mammary glands of their tranquilized five-year-old mother (Terri) on 15 May during the period of suckling (unpublished data). Although there are reports of yearlings suckling after emergence from dens in Montana [38] and Minnesota [11], we found no published reports of yearlings suckling in dens.

### 3.10. Water Intake, Urination, and Defecation by Yearlings and Adults

Although denning captive black bears have gone months without eating, drinking, urinating, or defecating [39,40], bears that denned for 5.1 to 7.7 months in this study urinated and defecated by backing out of the den bed to a latrine area near a den wall or out the entrance. On 3 March 2012, some of the urine that Jewel deposited at the den entrance flowed back into the den. On 1 April 2013, feces from Jewel’s yearling Fern rolled back into the bed area, where Jewel ate it. Their main sources of liquid were snow and icicles, plus the urine and feces that the mothers ingested as they toilet-licked newborn cubs. Mothers also licked drops of meltwater from the fur of their cubs. Prior to this study, three-year-old Blackheart licked drops of meltwater from the fur of her one-month-old cubs (Dot and Donna) when the temperature was 10 °C on 22 February 2000 [41]. Similarly, on 25 March 2007, six-year-old June licked water from her two-month-old cubs (Lily, Cal, and Bud) after an early melt (17 °C) temporarily flooded them from their den. On 19 September 2008 (16 °C), seven-year-old June licked water from the rock walls of a den she had occupied for 15 days.

### 3.11. Fecal Plugs 

The feces of late winter or early spring, harder and drier than summer feces, are often termed “fecal plugs”. This term may stem from an early belief that bears eat fibrous vegetation to plug the digestive tract and prevent further food intake during hibernation [29]. Fecal plugs contain little to no fibrous vegetation (unpublished data). When hydrated, they become a slimy, mucous substance [3]. Feces from hibernating bears may be similar to stools from humans with no food intake—primarily dead bacteria, enzymes, and desquamated cells [42]. The dryness of fecal plugs is presumably because they form over a period of months, giving the large intestine more time to remove water [42]. Feces from hibernating black bears can include pieces of foot pads (see below), hairs from grooming, bits of vegetation from raking, and the remains of body waste that mothers ingest from cubs during toilet-licking.

### 3.12. Eating Foot Pads

In January, adults and yearlings begin removing and ingesting old foot pads and exposing tender new ones [43]. In this study, mothers and yearlings used their teeth and claws to do this, with mothers sometimes aiding their yearlings.

### 3.13. Eye Opening

Eye opening was best viewed with Lily’s single cub (Hope), born on 22 January 2010. At 34 days of age on 25 February, Hope’s eyes were open 1–2 mm. Eight days later, at 42 days of age on 5 March, they were fully open (Table 2). Matson [9] reported a cub’s eyes to be partially open and a littermate’s fully open at 40 days of age on 12 February in Pennsylvania. Alt [15] found that the eyes of all 27 cubs he studied were fully open by 46 days of age in Pennsylvania.

### 3.14. Reciprocal Tongue-Licking

In our previous studies in northeastern Minnesota, we have seen cubs, juveniles, and adults touch tongues and engage in reciprocal tongue-licking in apparent signs of friendship (unpublished data), but we have not seen these behaviors reported for bears in dens. Reciprocal tongue-licking involves bears simultaneously touching and/or entwining tongues as they lick each other in and around the mouth (unpublished data). The webcams revealed Lily licking Hope’s mouth without Hope reciprocating when Hope was 39, 41, and 51 days old. However, on 17 March 2010, 54-day-old Hope vocalized the pulsing hum of suckling for 24 s during a 37-s session of reciprocal tongue-licking (Video S3). On 27 March 2010 during 16:55 to 17:04, 64-day-old Hope vocalized the pulsing hum throughout two sessions of reciprocal tongue-licking, lasting 5 min and 46 s and 2 min and 35 s, separated by a 41-s pause (Video S4).

### 3.15. Play

Up until now, observations of play development in bears has been restricted to post denning and hand-reared cubs [13,44,45]. The development of play behavior will be treated more thoroughly and quantitatively in these and other litters in a subsequent paper, but some interesting observations are highlighted here.

Lily’s two cubs (Eli and Ellie), born 12 January 2013, showed increasingly complex play as coordination developed. On 17 March 2013, the 64-day-old cubs batted feebly but playfully at each other and their mother (Video S5). On 31 March 2013, these 78-day-old cubs played more vigorously with each other. On 19 April 2013, 4 days before the family emerged, these 97-day-old cubs chased each other around and around Lily, sometimes slapping Lily’s face as they ran past.

Play by hibernating yearlings was less vigorous than the play of non-hibernating cubs. Play involving yearlings began on 14 February 2013 between Jewel’s 12-month-old yearlings Fern and Herbie and began on 13 February 2012 between four-year-old Lily and her 12-month-old daughter Faith. Play involving those yearlings was frequent until the bears emerged in spring.

### 3.16. Reactions to Large Mammals near Dens

When a moose foraged and broke branches outside Lily’s den on the night of 28 March 2011, Lily moaned in fear and repeatedly lunged, blew, and slapped toward the den entrance. She displayed similar behavior on the night of 11 March 2012 when wolves (*Canis lupus*), white-tailed deer (*Odocoileus virginanus*), and a fisher (*Martes pennanti*) passed within 30 m of the den, according to tracks found the next morning. Black bears in dens have been killed by wolves [11,46,47].

However, not all intrusions triggered overt responses. On 2 March 2013, a large dog barked at the entrance of Jewel’s den for nearly a minute while Jewel and her two yearlings appeared to continue sleeping, with Jewel breathing only 2.2 times per min—the slowest observed in this study. She had breathed 3.1 to 5.2 times per min (mean = 4.35, *n* = 4) during sleep or rest in the previous month.

### 3.17. Reactions to Small Mammals in Dens

Lily and Jewel sometimes reacted to small mammals that explored their dens or built nests in their bedding. On 3 January 2011, Lily raked a red-backed vole (*Clethrionomys gapperi*) nest out of her bedding, causing the vole to run out of the nest. The next evening (21:36 on 4 January 2011), a vole spent 11 min carrying material from the old nest ~25 cm to a spot where it presumably was making a new nest in Lily’s bedding. At first, Lily kept her head tucked under her chest, but a yearling, resting behind her, repeatedly raised its head and looked over her toward the vole. A moment later, Lily raised her head and laid it close to where the vole had been working. When the returning vole paused <15 cm from Lily’s nose, Lily turned her eyes toward it and the vole left.

On 2 February 2012, Lily jerked alert when a vole ran from under her hip. A week later, Lily slapped at a vole in her bed. On 28 December 2012, she looked toward a shrew (*Sorex* sp.) that had passed <7 cm from her head and slapped at it a minute later when it again passed near her. On 10 January 2013, she rose up and looked toward a vole that had run across the edge of her bed. On 16 January 2013, she blew sharply toward a vole that loitered <8 cm from her face after exploring her bedding for 21 s.

In contrast, Lily and her yearling (Faith) showed no response when a vole approached to <6 cm on 2 February 2012, and when a red squirrel (*Tamiasciurus hudsonicus*) approached just as close on 3 February 2012, after jumping down into the deep rock den and causing snow to fall onto the bears’ bed. Lily also showed no response on 12 January 2013, when a vole sniffed her head and another fell from the roof of her slash pile den onto her bed.

### 3.18. Reactions to Spiders and Snakes

At 16:06 on 16 February 2012, at least six unidentified spiders began moving about on the thick fur of Jewel’s side and back when afternoon temperatures reached 4 °C. She bit at them, possibly ingesting them. Although snakes are common in this area, may brumate in dens, and bears respond to them in varying ways [48], we saw no snakes in dens and are unaware of any ever being found in bear dens. 

### 3.19. REM Sleep and Dreaming

Newborn cubs frequently showed classic REM sleep with fluttering eyelids, randomly moving eyes, and twitching ears, legs, and faces. They occasionally showed full body (myoclonal) twitches [49]. REM sleep was less obvious in adults and yearlings. Their twitches were mainly facial, and they typically slept with their faces hidden. However, at 17:00 on 2 March 2012, four-year-old Jewel’s eyelids fluttered open, her eyeballs moved, and her muzzle and lips twitched and quivered. REM sleep has not previously been reported for bears of any species or age, in or out of dens. 

On 19 March 2013 at 02:46, Lily suddenly jerked awake as if from a dream, lunging and blowing straight ahead toward the wall of her den as if disoriented. Within a 6-s period (02:46:33–37), she oriented her display toward the den entrance and settled back to her cubs. A microphone at the entrance recorded no sound other than the bears, showing that no intruder was present. At 02:46:44, she raised her arm, yawned, and gathered her cubs to her to suckle. At 02:47:10, one of the cubs began the pulsing hum of suckling. Lily immediately put her head down as if she was no longer concerned. She then yawned, allowed her cubs to suckle, closed her eyes, and resumed her head-tucked sleeping position. REM sleep is purportedly associated with dreaming [50]. Prior to this study, three-year-old June, in REM sleep, vocalized the pulsing hum associated with suckling and contentment on 23 August 2004 (unpublished data).

## 4. Discussion

We have documented, in the natural dens of black bears, the occurrence of new behaviors and tracked the development of behavior. By using two female bears over multiple consecutive years, we showed both consistency and variation across denning events. For example, Jewel never raked objects into the den, whereas Lily always did so. We also have dens populated by cubs only, yearlings only, or both. Subsequent analyses of these and other videos can quantify the details of the interactions, but the present study indicates the wealth of data on these animals that can be obtained with bears conditioned to human presence as researchers, but not as companions.

The bears in this study were healthy and well nourished. Comparative studies are needed to determine how black bear behavior in dens varies with health, body condition, and region, all of which are known to affect black bear hibernation [11]. Across North America, hibernation schedules of black bears are adapted to regional differences in the annual cycles of plant growth and fruiting [37].

In northeastern Minnesota, the growing season is only 118 days, and denning periods are 156 to 235 days ([11], this study). Wild food is essentially absent for 7 months from October through April [9]. Bears in that region enter dens in September and October and do not remain active in response to garbage, bird feeders, or supplemental foods [11] (unpublished data). Bears that had been well nourished prior to hibernation responded quickly to disturbances during hibernation, but malnourished bears commonly did not arouse from hibernation until after several minutes of being disturbed, and they did not produce cubs. Mature females that weighed less than 67 kg on 1 October (*n* = 16) in northeastern Minnesota produced no cubs over winter, but those weighing more than 80 kg on that date gave birth in 28 out of 30 cases in which they had been without cubs the previous mating season [28,29]. A hibernating female without cubs conserved energy by shunting blood away from her legs [30].

By contrast, in southern states where growing seasons are longer and foods are available over winter, denning periods are only 6 to 142 days and some bears remain active throughout winter [51,52,53]. In the eastern deciduous forest, bears also remain active over winter when beechnuts or other foods are available ([53] and unpublished data).

Comparative webcam studies can help determine how denning behavior varies with body condition, region, and bear species. There are only eight bear species and all but the American black bear are threatened or endangered in much or most of their native habitat. Thus, behavioral research will aid conservation. Being intelligent and living in diverse habitats across much of their ranges, local variation in behavior at all life stages needs assessing. This is especially true as climate change can influence denning periods, food availability, seasonality, and mating.

Behavioral development of cubs in dens may be crucial to their survival after emergence. Being born in such an altricial state leads to the need to look at differences from wolves and dogs, for example, which are comparatively very well studied [20,21,22]. Even more so, however, we need comparative data among the various bear species, which are still sadly lacking, but which new technology may assist in studying. Over two decades ago, future directions in studying hibernating bears were proposed, including more comparative work [54]; behavior is a major component of such work and we have begun to correct misconceptions that were current not that long ago (54).

## 5. Conclusions

In this study we were able to directly observe the behavior of black bears in maternal dens. We recorded many aspects of their behavior, some of which have not been seen before; and which contradicted common views not based on such observation. The methods employed in the study should be useful in further studies of denning behavior in other species and populations of bears.

## Figures and Tables

**Table 1:** Den information for the six denning events.

Mother and Den	Burrow Dimensions L × H × W (cm)	Entrance Dimensions H × W (cm)	Date of Final Ingress	Date of Final Egress
**Lily 1**	77 × 41 × 65	34 × 77	3 October 2009	1 April 2010 (180 days)
**Lily 2**	173 × 55 × 87	55 × 53	19 October 2010	8 April 2011 (171 days)
**Lily 3**	180 L × 60–90 W	36 × 72	20 October 2011	24 March 2012 (156 days)
**Lily 4**	150 × 90 × 120	Irregular slash pile	31 August 2012	23 April 2013 (235 days)
**Jewel 1**	180 × 29 × 72	29 × 72	Mid-October	12 April 2012 (173–183 days)
**Jewel 2**	266 × 58 × 72	55 × 55	9 October 2012	27 April 2013 (200 days)

L: length, H: height, W: width.

**Table 2:** Mother and offspring demographic and developmental data for the six litters of black bears observed in their dens in Minnesota.

Mother	Mother’s Age	Yearlings	Cubs: Sex	Genital Swelling	Date of Birth	Eyes Confirmed Fully Open
**Lily 1**	33 months	None	Hope: F	11 January 2010	22 January 2010	5 March 2010 (42 days)
**Lily 2**	45 months	Hope (9 months)	Faith: F Jason: M	16 January 2011	21 January 2011	22 February 2011 (32 days)
**Lily 3**	57 months	Faith (9 months)	None			
**Lily 4**	69 months	None	Eli: M Ellie: F	11 January 2013	12 January 2013	19 February 2013 (38 days)
**Jewel 1**	33 months	None	Fern: F Herbie: M	19 January 2012	22 January 2012	7 March 2012 (38 days)
**Jewel 2**	45 months	Herbie, Fern (9 months)	None			

## Supplementary Materials

The following video clips are available online. Video S1: three-year-old Lily licking her swollen birth canal prior to parturition on 22 January 2010, https://youtu.be/5bqvAD8vDLw, Video S2: six-year-old Lily toilet-licking her 52-day-old cub on 5 March 2013, https://youtu.be/jq2-EATMK3w, Video S3: six-year-old Lily and her 54-day-old cub doing reciprocal tongue-licking on 17 March 2010, https://youtu.be/eax-SVuu2_c, Video S4: six-year-old Lily and her 64-day-old cub doing reciprocal tongue-licking on 27 March 2010, https://youtu.be/xBMzikv0AHE, Video S5: six-year-old Lily playing with her 64-day-old cubs on 17 March 2013, https://youtu.be/9Ybg79eQQdA.
